# Comparing a 7-Day Food Diary and Repeated 24-Hour Dietary Recall for Estimating Usual and Operational Definitions of Acute Intake in Danish Adults

**DOI:** 10.3390/nu18121845

**Published:** 2026-06-08

**Authors:** Anja Biltoft-Jensen, Juan J. Aristizabal-Henao, Ken D. Stark, Tue Christensen

**Affiliations:** 1 National Food Institute, Technical University of Denmark, 2800 Kongens Lyngby, Denmark; tuchr@food.dtu.dk; 2 BPGbio, BPGbio Inc., Waltham, MA 02451, USA; juan.henao@uwaterloo.ca; 3 Department of Kinesiology and Health Sciences, University of Waterloo, Waterloo, ON N2L 3G1, Canada; kstark@uwaterloo.ca

**Keywords:** dietary assessment, 7-day food diary, 24-h dietary recall, usual dietary intake, acute dietary intake, fish intake, EPA, DHA, whole-blood biomarkers, validation

## Abstract

**Background:** Accurate dietary assessment is essential for estimating habitual and acute exposures. This study compared a 7-day food diary (7dFD) and repeated 24-h dietary recall (2×24hDR) for habitual and acute intake. Habitual fish and *n*-3 PUFA estimates were evaluated against whole-blood EPA and DHA, and acute intake of fish, rye bread, and coffee was explored using operational indicators. **Methods:** In a within-subject comparative design, 120 Danish adults aged 18–60 years completed both methods. Fish and *n*-3 fatty acid intakes were compared with whole-blood EPA and DHA. Usual fish intake from 2×24hDR was estimated using the Multiple Source Method (MSM) with Food Propensity Questionnaire (FPQ) data. Acute intake was assessed using single-day, consumption-day, single-meal, maximum-day, and meal-weighted estimates. **Results:** EPA, DHA, total fish, and fatty fish intakes estimated from the 7dFD and MSM-adjusted 2×24hDR correlated with biomarkers r = 0.23–0.46. Supplement inclusion improved EPA and DHA correlations (r = 0.26–0.59). The unadjusted 2×24hDR identified fewer fish consumers than the 7dFD (63% vs. 88%), whereas FPQ-based modelling identified 97%. Acute intake estimates varied by method and definition: the 2×24hDR produced higher short-term and upper- percentile estimates, while the 7dFD produced more stable distributions. Limitations include modest sample size, upper-percentile uncertainty, and non-equivalent supplement assessment. **Conclusions:** In this adult convenience sample, the 7dFD provided more stable habitual intake estimates, whereas the 2×24hDR produced higher short-term and upper-percentile estimates under the applied operational acute-intake definitions. These findings are context-dependent and should not be taken as evidence that either method is generally superior.

## 1. Introduction

The Danish National Food Institute, like other institutions and authorities, relies on dietary intake data to assess nutritional status, food safety, and climate impact. The Food Institute has used a 7-day food diary (7dFD) to estimate dietary intake for many years. However, the European Food Safety Authority (EFSA) has suggested that all member countries use the 2 × 24-h dietary recall (2×24hDR) method. The 2×24hDR has shown accurate results for estimated energy intakes in a subgroup of Danish adults compared to doubly labeled water [1,2]. Since 2×24hDR only captures intake on two random days, it may underestimate consumption if a food is not recorded or overestimate peak intake if a high-consumption day is included. This is particularly problematic in exposure assessments, where upper percentiles are used to evaluate acute and chronic risks. Combining 2×24hDR with statistical modeling and food frequency data can help address these limitations.

Fish is an episodically consumed food in Denmark and an important source of eicosapentaenoic acid (EPA) and docosahexaenoic acid (DHA), iodine, other micronutrients, and high-quality protein [3,4,5]. At the same time, fish may contribute to exposure to environmental contaminants, making both habitual and high-end intake estimates relevant for dietary risk–benefit assessment [3,6].

EPA and DHA can be used as objective indicators of fish intake because their endogenous synthesis from their precursor alpha-linolenic acid (18:3*n*-3) is low, and fish and seafood are the primary dietary sources [7,8]. EPA and DHA content in whole blood (mixture of plasma and erythrocytes) has previously been used as biomarkers of EPA, DHA, and fish intake [7,9,10].

For many diet-health and nutrient-adequacy questions, usual intake is the primary exposure of interest. In contrast, food-safety assessments may also require estimates of short-term or high-end intake.

The 7dFD and 2×24hDR are short-term methods, with 2×24hDR being the shortest, and they may not accurately capture long-term consumption. Several statistical models have been developed to mitigate this limitation and reduce intra-individual variance when estimating chronic exposure, especially from 2×24hDR. Food consumption data collected over two non-consecutive days, combined with statistical modeling, can assess the usual intake distribution of a population [11,12,13]. However, there has been less focus on estimating acute intake, which is relevant for exposure assessment of contaminants and other substances, such as coffee and caffeine intake. Suitable estimation of acute intake is vital for protecting public health, ensuring regulatory compliance, managing risks, and supporting informed decision-making in both public health policy and the food industry.

Acute estimates depend on the substance, as a single dose might elicit an adverse effect (e.g., caffeine), or a cumulative dose over several days might be necessary before an effect is observed (e.g., fish and heavy metals or PCBs).

Health organizations such as EFSA, FDA, FAO, and WHO have described acute consumption as lasting no longer than a day [12,14,15,16,17]. There is no single universally accepted operational definition of acute food intake. In exposure assessment, acute intake is commonly assessed over a short period, such as a single eating occasion or a single day, depending on the substance, food, and risk question. Consequently, different operational definitions may produce different intake distributions and upper-percentile estimates.

Previous validation studies have evaluated dietary records, 24-h recalls, food frequency instruments, and statistical usual-intake models for selected nutrients or food groups. However, less is known about how a 7dFD and 2×24hDR compare head-to-head in the same adult sample when evaluated simultaneously for biomarker-supported usual intake and for several operational definitions of acute intake. This distinction is important because the preferred method may depend on whether the objective is to estimate habitual intake, consumer status, upper percentiles, single-day intake, single-meal intake, or maximum short-term exposure.

The main objective of this study was to compare the 7dFD and 2×24hDR within the same Danish adult sample for estimating habitual and acute dietary intake. As a validation component, we evaluated the relative validity of habitual fish and *n*-3 PUFA intake estimates against whole-blood EPA and DHA biomarkers. As an exploratory methodological component, we compared observed acute-intake distributions for fish, rye bread, and coffee using several predefined operational indicators: average intake across observed days, intake on consumption days, single-meal intake, maximum-day intake, and meal-weighted intake. The purpose was not to identify a single definitive acute-intake estimator, but to discuss how different indicators serve different exposure-assessment questions.

## 2. Research Design and Methods

### 2.1. Study Design and Population

The study was conducted at the National Food Institute (NFI), Technical University of Denmark as part of evaluating two dietary assessment methods. The overall study design, recruitment procedures, and data collection have been described in detail elsewhere [1]. Briefly, 120 volunteers aged 18 to 60 years were recruited in the age groups 18–30, 31–45, and 46–60 years to capture the different life stages in the adult population. The study was designed for methodological comparison and validation rather than estimation of population-level intake distributions. The sample size was determined for the parent validation study of energy intake against doubly labeled water [1]. The present analyses of fish intake, *n*-3 PUFA biomarkers, and acute-intake percentiles were secondary and were not covered by a separate a priori power calculation.

All participants underwent an in-depth telephone screening interview. To be included, participants had to speak Danish, have internet access, agree to the study task, have basic good health, be weight stable, and not actively trying to lose weight or taking medications known to affect food intake or appetite. Pregnant and lactating females and nutrition professionals were also excluded.

**Figure 1:**
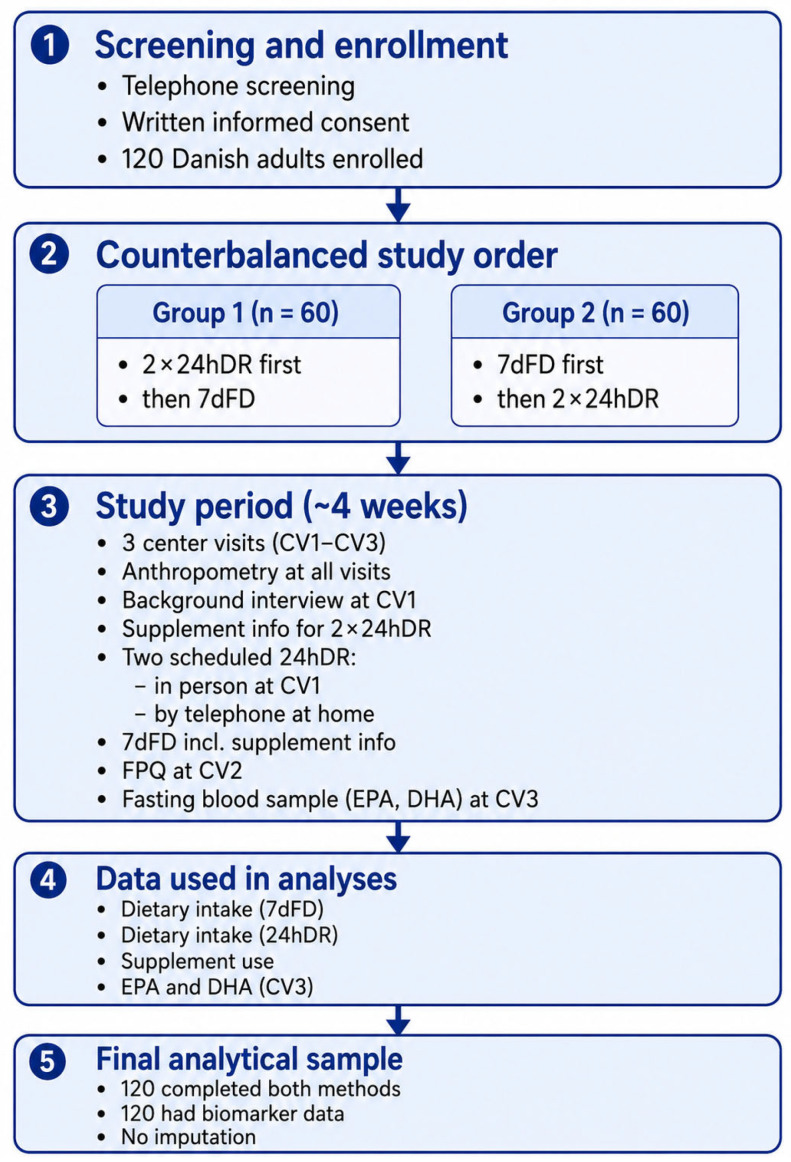
Study flow and data collection (7dFD, 7-day food diary; 2×24hDR, two 24-h dietary recalls; CV, center visits).

The study used a within-subject comparative design in which all participants completed both dietary assessment methods in a counterbalanced order; 60 started with 24hDR and 60 with 7dFD. The data collection flow is shown in Figure 1. Data was collected during September and October 2017.

Each participant took part for approximately four weeks, including three center visits (CV), two scheduled 24hDR interviews (one in-person and one by telephone), seven days of dietary recording. Blood samples were collected on the final visit to the center. Anthropometric measurements of weight, and body composition were taken at all three CV. Weight and body composition were measured by bioelectrical impedance analysis using a Tanita BC-418 MA body composition analyser (Tanita Corporation, Tokyo, Japan), with participants wearing light clothing and no shoes. At CV1, all participants had their height measuredto the nearest 0.1 cm using a wall-mounted stadiometer (KERN MSF 200; KERN & SOHN GmbH, Balingen, Germany). Two measurements were performed for each participant. If the two measurements differed by more than 1 cm, a third measurement was taken, and the mean of the three measurements was used. Furthermore, social background characteristics were assessed through a short interview.

A personal 24hDR was conducted at CV1 or CV2 by trained interviewers, and detailed instructions on recording dietary intake in the 7dFD were provided individually to all participants at CV1 or CV2 depending on the starting method. The blood sampling was completed at CV3. The 2×24hDR were completed on randomly selected weekdays, covering all seven weekdays at the group level and at least one week apart, as recommended by EFSA [12]. Everyone entering the study provided written informed consent. The study was conducted in accordance with the Declaration of Helsinki and the protocol approved by the Research Ethics Committees of the Capital Region, Denmark (H-17006825) on (2 May 2017). The study was not registered as a clinical trial, as its primary aim was methodological comparison and validation of dietary assessment methods rather than evaluation of the effect of a health-related intervention on a health outcome.

### 2.2. 24 H Dietary Recalls—Automated Multiple Pass Method (AMPM)

Dietary recalls were conducted using a Danish version of the USDA Automated Multiple Pass Method (AMPM), a structured five-step interview approach designed to improve recall of foods, amounts, eating occasions, and preparation details [2].

Six trained interviewers with formal nutrition-, public health- or biology education backgrounds conducted all 24hDR interviews.

Because the AMPM does not include questions about supplements, supplement use was assessed during the in-person background interview before the first 24 hDR. Participants reported use of vitamin/mineral supplements, fish oil, or other supplements during the previous year, including frequency, brand name, and dose per administration.

### 2.3. Food Propensity Questionnaire

A web-based FPQ was utilized to evaluate the intake of primarily episodically consumed foods and beverages, following recommendations from EFSA [12]. It comprised approximately 36–57 questions, depending on the answers, regarding the consumption of 36 food groups including beverages, bread, fish, meat, and vegetables.

The FPQ asked about dietary habits over the previous year, focusing on consumption frequency. Frequency categories ranged from never to two or more times per day.

### 2.4. 7-Day Food Diary

Participants recorded their food intake each day for 7 consecutive days in the 7dFD. The 7dFD was a web-based diary covering six daily eating occasions. Foods were selected from a database or entered as free text, and portion sizes were estimated using digital portion-size images. The diary included internal checks for commonly forgotten foods.

At the end of the diary registration respondents were asked to register their daily intake of dietary supplements, including vitamins and minerals, fish oil and other supplements. Participants could choose among the 12 best-selling brands of multi-vitamin/mineral and fish oil supplements or write an open-ended answer. Participants were not asked about the dose, as it was assumed that they had taken the dose recommended by the producer. To be included in the analyses, participants had to complete the 7dFD for at least 4 days, including one weekend day to represent a weekly recording. The 7dFD and 2×24hDR have previously been validated for estimated energy intake in Danish adults using doubly labelled water [1]. Fish including shellfish were classified as lean fish (<4 g fat/100 g; e.g., cod, shrimp, and flounder) or fatty fish (≥4 g fat/100 g; e.g., salmon, herring, and mackerel). Participants were categorised as fish oil supplement users if they took *n*-3 long-chain PUFA-containing supplements at least once during the week. Supplement intake was assessed differently across methods. In the 7dFD, participants reported supplement use during the diary period, whereas supplement use linked to the 2×24hDR was assessed using longer-term frequency information from the background interview. For both methods the nutrient intake was calculated for each individual with the use of the software system GIES (Version 1.000 i5—10 September 2014), developed at the National Food Institute, Technical University of Denmark, and the Danish Food Composition Databank Frida (version 3; Soeborg, Denmark; 23 March 2018).

### 2.5. Fatty Acids Biomarkers

A fasting venous blood sample was drawn from the antecubital vein at CV 3. Heparinised blood was mixed with 0.1% butylated hydroxytoluene (BHT; Sigma-Aldrich, St. Louis, MO, USA), in ethanol (0.1 mL/mL blood) and stored at −80 degrees C. Whole-blood fatty acid composition was measured by a high-throughput gas chromatographic method as described in detail previously [18]. Briefly, fatty acid methyl esters were prepared from whole blood by direct transesterification with 14% BF_3_ in methanol using convectional heating (95 degrees C for 1 h). Fatty acid methyl esters were analysed by GC with flame ionization detection using a Varian 3900 (Varian Inc., Palo Alto, CA, USA) equipped with a DB-FFAP 15 m × 0.10 mm inner diameter × 0.10 µm film thickness column. Fatty acids were identified by comparison to an external mixed standard (GLC-462; Nu-Chek Prep, Inc., Elysian, MN, USA), and absolute concentrations of individual fatty acids (μg of fatty acid/100 µL of whole blood) were determined by comparison with an internal standard added before transesterification (22:3*n*-3 ethyl ester; Nu-Chek Prep, Inc., Elysian, MN, USA),). The full fatty acid composition of the participants has been published previously [9]. Biomarkers of *n*-3 PUFA were expressed as composition by weight percentage of total fatty acids (*w*/*w*%). The inter- and intra-assay CV were 4.5 and 1.2% (EPA) and 6.4 and 2.4% (DHA). The limit of quantification for the fatty acids ranged between 0.007 and 0.01 µg/100 µL as determined by calibration curves.

### 2.6. Statistical Method

All 120 participants included in the present analyses completed both dietary assessment methods and had available biomarker data. Therefore, no imputation of missing dietary or biomarker data was performed. Non-consumption of food on recorded days was treated as zero intake in analyses that included zero-intake days. Because there was substantial difference in energy intake between the two dietary assessment methods, the relative intake (g/10 MJ) has been used for the analysis when comparing usual intake of fish, to reduce the influence of systematic differences in reported energy intake. Acute intake estimates were not energy-adjusted because acute dietary exposure assessment is typically based on absolute short-term intake.

The dietary intake of individual fatty acids was transformed to a percentage of total fat intake to make them comparable to the weight percentage of individual fats in whole-blood. Hence, it is total fatty acid intake distributed into individual fatty acids that are compared. The normality of variables was tested using the Shapiro-Wilk test. Most fatty acids and fish intake were not normally distributed. Spearman’s rank partial correlation was used to correlate fatty acids (*w*/*w*%) as it does not assume normality.

To estimate usual intake, we applied the Multiple Source Method (MSM), a statistical modeling approach designed to adjust for within-person variation and episodic consumption in dietary intake [13,19]. The MSM model included age, sex, and age × sex as covariates. Sex-stratified biomarker correlations were exploratory, with no formal tests of sex differences in correlation coefficients. Although methods were completed in counterbalanced order, period, sequence, and order effects were not formally modeled.

For usual fish intake from the 2×24hDR, the MSM model incorporating FPQ frequency information was considered the primary modeling scenario because fish is episodically consumed and the FPQ provides longer-term information on consumption probability. Frequencies of fish intake were converted to a continuous variable, assuming 30.4 days in a month (Table 1). MSM scenarios without FPQ information or with alternative assumptions about the proportion of consumers were retained as sensitivity analyses to illustrate the dependence of usual intake estimates on modelling assumptions. The MSM method was selected over the NCI because it accounts for within-person variability while remaining computationally feasible with the present sample size.

Five operational indicators of acute intake were evaluated as descriptive analytical scenarios. They were not intended to represent standardized or validated acute-exposure methods: (a) mean intake across all recorded days among consumers, including zero-intake days; (b) mean intake on consumption days only; (c) intake per single meal among meals with consumption; (d) maximum intake on one consumption day; and (e) meal-weighted intake, assigning higher weights to meals considered more likely to represent high-intake occasions for the specific food. The interpretive framework for the acute-intake indicators used in this study is presented in Table 2. The meal-weighted indicator was included as an exploratory sensitivity analysis. A weighting factor of 1.15 was applied to selected meals to reflect the approximate magnitude of under-reporting previously observed for the 7dFD in the parent validation study against doubly labeled water [1]. This factor was not derived specifically for fish, rye bread, coffee, or individual meal occasions. Therefore, the meal-weighted estimates should not be interpreted as corrected intake estimates, but as an exploratory operational scenario examining the effect of giving greater weight to meals considered more likely to contain higher intakes of the food in question. Weighting factors are shown in Table 3, and formulas are provided in Appendix A.

## 3. Results

### 3.1. Characteristics of the Study Population

120 participants, with a mean age of 39 years, completed the study including 2×24hDR and all 7dFD. General characteristics of the study population are presented in Table 4. The sample was relatively highly educated, with 64% having medium or long higher education. Thirty-eight percent had a BMI above 25 kg/m^2^. Sixteen percent were classified as using fish oil supplements within the last week in the 7dFD. For the 2×24hDR 31% were classified as fish oil supplement users with at least periodical intake during the previous year. Reported energy intake was higher with the 2×24hDR than with the 7dFD: 11.6 MJ/day compared with 9.5 MJ/day.

### 3.2. N-3 PUFA and Fish Intake vs. Blood Biomarker EPA, DHA

Table 5 shows that estimated intakes of *n*-3 PUFA and fish from both the 7dFD and the MSM-adjusted 2×24hDR were significantly correlated with whole-blood EPA and DHA biomarkers, with correlation coefficients ranging from 0.23 to 0.46 for diet-only estimates. Correlations were generally stronger for the 7dFD than for the MSM-adjusted 2×24hDR. Including method-specific supplement information increased the correlations, particularly for EPA and DHA, with the highest correlations observed for EPA + DHA including supplements. For example, EPA + DHA including supplements correlated with biomarkers at r = 0.59 for the 7dFD and r = 0.38 for the MSM-adjusted 2×24hDR.

Total fish and fatty fish intake correlated significantly with biomarker EPA + DHA from both 7dFD and 2×24hDR adjusted by the MSM and consumption frequency. Correlations were weaker or non-significant for lean fish. Correlations appeared stronger among men than women, but sex differences were not formally tested and should be interpreted descriptively.

Partial Spearman’s rank correlation between fish intake estimated by the FPQ using standard portions and blood biomarker EPA + DHA, controlled for age, gender, and education also showed significant correlations, r = 0.32 overall (*p* < 0.001).

### 3.3. Estimating Usual Intake

Table 6 presents the estimated usual fish intake. The estimated usual fish intake differed by dietary assessment method and modelling scenario. The proportion classified as fish consumers was 88% with the 7dFD, 63% with the unadjusted 2×24hDR, and 97% with the FPQ. The MSM + FPQ model, considered the primary model-based usual-intake scenario, identified 97% as consumers and estimated a mean fish intake of 33 g/10 MJ. Mean fish intake was broadly similar for the 7dFD and the 2×24hDR-based MSM scenarios, but upper percentiles differed across modelling assumptions. For example, the 95th percentile was 84 g/10 MJ for the 7dFD, 134 g/10 MJ for the unadjusted 2×24hDR, and 63 g/10 MJ for the MSM + FPQ scenario.

### 3.4. Effect of Sampling Days and Periods

Table 7 presents differences in food intake (g/day) when based on different sampling days and periods by seven-day food diary. Intake estimates varied by sampling strategy and food type. Fish showed the greatest variation in consumer proportions, ranging from 34–51% on single-day estimates to 88% across the full 7dFD. For fish, the population mean was 22 g/day on one random day, 28 g/day based on two random days, and 26 g/day across all seven days showing that fish intake based on two random days was close to the full 7dFD estimate. For coffee, corresponding means were 321, 326, and 323 g/day, and consumer proportions of 64–75% across single- and two-day strategies and 78% across the full 7dFD indicating more stable estimates across sampling periods. Rye bread showed an intermediate pattern, with consumer proportions ranging from 48–70% across single- and two-day sampling strategies and 83% across the full 7dFD.

Across all foods, single-day sampling tends to underestimate both the proportion of consumers and total intake, especially for episodically consumed items like fish and rye bread. These findings emphasize the need for multi-day assessments or meal-specific correction strategies when estimating acute or high-percentile intake.

### 3.5. Estimating Acute Intake

Table 8 presents estimates of five different operational definitions of acute intake for fish, rye bread, and coffee based on the 7dFD: daily intake including zero-intake days, daily intake on consumption days only, single-meal intake, maximum intake on a single day, and meal-weighted daily intake. Each detailing the number of consumers, mean intake, and percentile values from the 1st to the 99th percentile.

Estimates generally increased when moving from all observed days to consumption-day, single-meal, maximum-day, and meal-weighted indicators. For fish, mean intake increased from 29 g/day when zero-intake days were included to 108 g/day for maximum-day intake, while the corresponding p95 increased from 70 to 258 g/day. The increase was most pronounced for fish and rye bread, reflecting greater variation between days and eating occasions. Coffee showed the highest absolute intakes, with mean maximum-day intake of 659 mL/day. The meal-weighted estimates are included only as an exploratory sensitivity scenario and should not be interpreted as a validated exposure-estimation method.

Table 9 presents estimates of different operational definitions of acute intake for fish, rye bread, and coffee based on 2×24hDR: daily intake including zero-intake days, daily intake on consumption days only, single-meal intake, maximum intake on a single day, and meal-weighted daily intake. Each detailing the number of consumers, mean intake, and percentile values from the 1st to the 99th percentile.

As for the 7dFD, estimates generally increased when moving from all observed days to consumption-day, single-meal, maximum-day, and meal-weighted indicators. Fish showed marked variation across indicators, and mean intake increased from 68 g/day when zero-intake days were included to 121 g/day for maximum-day intake, while the corresponding p95 increased from 188 to 369 g/day. Rye bread showed a more even distribution. Coffee showed the highest absolute intakes, with mean maximum-day intake of 853 mL/day. The meal-weighted estimates are included only as an exploratory sensitivity scenario and should not be interpreted as a validated exposure-estimation method.

## 4. Discussion

The present study should not be interpreted as identifying a definitive “true” method for estimating acute dietary exposure. Rather, it compares the observed intake distributions generated by two dietary assessment methods under several operational definitions of acute intake. These operational definitions were selected for comparison in this study and should not be interpreted as standardized or validated methods for acute exposure assessment. Each definition reflects a different exposure question: average intake across observed days, intake on consumption days, single-meal intake, maximum daily intake, and, as an exploratory sensitivity scenario, a meal-weighted intake. Consequently, differences between the 7dFD and 2×24hDR reflect both the dietary assessment instrument and the analytic definition applied.

### 4.1. Validating Dietary Assessment of N-3 Fatty Acids and Fish Using 7dFD and 2×24hDR

Accurate intake data are essential for dietary risk assessments, particularly for critical nutrients like EPA, DHA, and fish. In this study, we evaluated the relative validity of two dietary assessment methods, 7dFD and 2×24hDR, against whole blood biomarkers of *n*-3 fatty acids. Both 7dFD and 2×24hDR showed significant correlation coefficients ranging from 0.26 to 0.55, with the 7dFD generally producing higher correlations. DHA and EPA showed the highest correlations for the 7dFD (0.55 and 0.51), whereas the 2×24hDR showed greater variability (0.26 and 0.36). When method-specific supplement information was included, EPA and DHA correlations increased consistently with the contribution of regular supplement use to longer-term whole-blood *n*-3 PUFA levels. Because supplement data were collected differently across methods, these supplement-inclusive correlations should be interpreted as method-specific sensitivity analyses rather than strictly equivalent comparisons. Because the recall method captured intake over only 2 days, it may be less aligned with longer-term whole-blood biomarker levels than the 7dFD, particularly for episodically consumed foods.

These findings align with earlier reviews by Serra-Majem et al. (2012) and Øverby et al. (2009), which reported correlations of 0.40–0.60 when validating FFQs, weighed records, and diet histories for *n*-3 fatty acid intake, though without using whole blood biomarkers [20,21]. Studies using whole blood, such as Shen et al. (2019), found higher correlations than ours (0.50–0.71) [22].

### 4.2. Estimating the Usual Intake of Episodically Consumed Foods Such as Fish

Estimating the usual intake for episodically consumed foods like fish is methodologically challenging because consumer classification and upper percentiles are highly sensitive to the number of observation days and assumptions about consumption probability. In this study, the proportion classified as fish consumers varied from 63% with the unadjusted 2×24hDR to 88% with the 7dFD and 97% with the FPQ. The MSM + FPQ model was considered the primary model-based usual-intake scenario because the FPQ provided longer-term frequency information; however, the range across MSM scenarios shows that the estimates were sensitive to modeling assumptions. MSM adjustments reduced the upper tail of the fish-intake distribution, for example, the 95th percentile decreased from 134 g/10 MJ/day in the unadjusted 2×24hDR to 63 g/10 MJ/day in the MSM + FPQ scenario. This is consistent with previous comparisons of usual-intake models, which show that statistical adjustment can shrink intake distributions and may attenuate high-percentile estimates [11,23]. A key challenge arises when methods like the MSM assume that the transformed usual intake follows a normal distribution. If the transformed intake remains skewed, this can lead to “overshrinking,” particularly at the tails of the distribution, artificially reducing variability [24,25]. Thus, MSM improves estimation of usual intake distributions but requires cautious interpretation when high-end intake is relevant for risk assessment.

### 4.3. Effect of Sampling Days and Periods

The comparison of sampling days and periods illustrated how food consumption patterns influence intake estimates. Coffee, a habitually consumed beverage, showed relatively stable mean intakes across sampling strategies. In contrast, fish showed greater variation in consumer proportions and mean intake depending on the selected day or period. Rye bread showed an intermediate pattern. These findings support the general principle that foods consumed episodically require longer observation periods or supplementary frequency information. In contrast, more habitual foods are less sensitive to the number and timing of recording days. Two non-consecutive random days approximated the 7dFD mean intake for fish at the population level, but percentile estimates remained more sensitive to sampling design.

This supports earlier findings by Larkin et al. (1999) and EFSA recommendations that random, non-consecutive sampling improves representativeness and reduces bias, particularly for foods with irregular intake patterns [12,26].

### 4.4. Acute Consumption

The observed acute-intake distributions varied substantially depending on the operational definition used. The indicators evaluated here were not intended to represent standardized acute-exposure methods, but were predefined analytical scenarios used to illustrate how different short-term exposure questions affect the observed intake distribution. Estimates including zero-intake days described average intake across observed days among consumers, whereas consumption-day, single-meal, and maximum-day estimates described progressively more selected high-intake scenarios. These estimates should therefore be interpreted as complementary descriptions of different short-term exposure questions, not as competing estimates of one true acute exposure distribution.

For episodically consumed foods such as fish, excluding non-consumption days or focusing on single meals or on maximum-day intake increased mean intake and widened upper percentiles. This pattern was more pronounced for the 2×24hDR, because fewer observation days made the distribution more sensitive to whether a high-intake event occurred on a recall day. For more habitually consumed foods, such as coffee, the differences between definitions were smaller for mean intake but remained relevant at the upper percentiles.

The use of a meal-weighted approach was included as an exploratory operational definition. It illustrated how weighing selected eating occasions can influence high-intake estimates, but it should not be interpreted as a standardized or validated acute-exposure method, nor as a corrected intake estimate.

### 4.5. Comparison Between 2×24hDR and 7dFD

Overall, the comparison between the 2×24hDR and 7dFD illustrates that method characteristics and analytic definitions jointly shape the observed intake distributions. The 2×24hDR produced higher upper-percentile and maximum-intake estimates for several foods, reflecting greater sensitivity to high-intake events occurring on recall days. The 7dFD produced more stable distributions across the week, but this stability may attenuate short-term peaks. These differences do not indicate that one method provides a more valid estimate of acute exposure; rather, they show that each method answers different exposure questions.

Reported energy intake was higher with the 2×24hDR than with the 7dFD. Energy-adjusted estimates were therefore used for usual fish intake, whereas acute intake was retained on an absolute scale because acute exposure assessment concerns the amount consumed over a short period. However, differences in reported energy intake may have contributed to the observed between-method differences in acute-intake distributions.

The interpretation of these operational indicators depends on the food’s consumption pattern and the risk-assessment question including whether the focus is usual intake, average short-term intake, observed high-end intake, or single-eating-occasion exposure.

### 4.6. Strengths and Limitations

A key strength of this study is its integrated design, combining multiple dietary assessment tools, 7dFD, 2×24hDR, and an FPQ, with objective whole blood biomarkers EPA and DHA. This multi-method approach enabled comparison of both habitual and acute intake estimates within the same adult sample. The 7dFD provided a continuous intake record over a full week, whereas the 2×24hDR offers in-depth, meal-level data, relevant for assessing short-term intake and within-day variability. The FPQ complements both methods by accounting for longer-term dietary patterns, especially for episodically consumed foods like fish.

Several limitations should be considered. First, participants constituted a convenience sample of Danish adults and were not selected to be nationally representative. The findings should therefore be interpreted primarily as within-sample method-comparison results rather than as population-level estimates of Danish intake distributions.

Second, the analyses of fish intake, *n*-3 PUFA biomarkers, and acute-intake percentiles were secondary and were not covered by a separate a priori power calculation. The modest sample size limited precision, particularly for biomarker correlations, sex-stratified analyses, and upper-percentile estimates. Sex-stratified findings and 99th percentile estimates should therefore be interpreted as exploratory and descriptive.

Third, although the data collection was organized in a counterbalanced within-subject design, period, sequence, and order effects were not formally modeled. Differential reactivity or order-related effects cannot be excluded.

Fourth, supplement intake was not captured equivalently across methods, limiting the direct comparability of supplement-inclusive EPA and DHA estimates. Similarly, self-reported dietary data may be affected by memory limitations, errors in portion-size estimation, respondent burden, and social desirability bias.

Reported energy intake differed substantially between methods, and no formal energy-plausibility analysis was conducted.

Finally, MSM-based usual-intake estimates depend on modeling assumptions and may be sensitive to sample size, especially for episodically consumed foods and upper-percentile estimates [23]. Therefore, MSM-adjusted high-end intake estimates should be interpreted cautiously.

## 5. Conclusions

In this convenience sample of Danish adults, the 7dFD and 2×24hDR produced different estimates of habitual and acute intake, depending on the food, dietary assessment method, and operational intake definition applied. Biomarker correlations supported the use of both methods for ranking habitual fish and *n*-3 PUFA intake, although correlations varied by method and were influenced by supplement inclusion.

For acute intake, the 2×24hDR generated higher upper-percentile and maximum-intake estimates, whereas the 7dFD produced more stable distributions across a longer observation period. These differences should be interpreted as method- and definition-dependent rather than as evidence that one method provides a definitive estimate of acute exposure.

For episodically consumed foods like fish, method choice has a pronounced impact on consumer classification, percentile estimates, and the interpretation of high-end intake. Statistical adjustment using MSM helped estimate usual intake distributions but may also have compressed upper percentiles. Consumer-day, single-meal, and maximum-intake analyses provided complementary descriptions on short-term intake.

Overall, the operational acute-intake indicators should be viewed as descriptive analytical scenarios within this study, not as standardized or validated methods for acute exposure assessment; their interpretation depends on the exposure question, the food’s consumption pattern, and whether the assessment prioritizes habitual intake, observed high-end short-term intake, or single-eating-occasion exposure.

## Figures and Tables

**Table 1:** Frequencies of Fish Intake.

Category	Mid_Interval	Frequency_per_Day
Never	0	0.000
1 or less per month	0.5/30.4	0.016
2–3 per month	2/30.4	0.066
1–3 per week	2/7	0.286
4–6 per week	5/7	0.714
1 per day	7/7	1.000
2 or more per day	1 × 2	2.000

**Table 2:** Interpretative framework for acute-intake indicators.

Indicator	Main Exposure Question	Best Suited for	Main Limitation
(a) Mean across observed days, including zero-intake days	What is the average short-term intake among consumers across observed days?	Population-level description, including non-consumption days	Dilutes intake for episodically consumed foods
(b) Consumption-day intake	What is intake when the food is consumed?	Foods with episodic consumption; consumer-day exposure	Excludes non-consumption days and therefore describes a selected scenario
(c) Single-meal intake	What is the intake per eating occasion?	Substances where risk may relate to one eating occasion	Does not represent full-day intake
(d) Maximum daily intake	What is the highest observed daily intake?	Conservative description of observed high-intake days	Sensitive to outliers and the number of observation days
(e) Meal-weighted intake	How does weighting selected eating occasions affect high-intake estimates?	Exploratory sensitivity analysis for meal-related intake variation	Not an established acute-exposure metric; method-specific

**Table 3:** Food Weight Factors Meals.

Meal	Fish	Ryebread	Coffee
breakfast	1.00	1.00	1.15
lunch	1.15	1.15	1.00
dinner	1.15	1.00	1.00
snacks	1.00	1.00	1.15

**Table 4:** Characterization of the study population.

Study Population (*n* = 120)	All	Males	Females
Sex (%)	-	43	57
Age (years)	39 (12)	37	40
Weight (kg)	73.0 (12.3)	81.5	67.3
Underweight (%)	2	0	3
Normal weight (%)	60	52	66
Overweight (%)	33	44	25
Obese (%)	5	4	6
In the process of education (%)	19	25	15
Short education (12–13 years) (%)	17	17	16
Medium higher education (15–17 years) (%)	19	16	22
Long higher education (+17 years) (%)	45	42	47
Smoking daily, at least once a week or occasionally (%)	8	8	9
Taking fish oil supplement at least once the week of registration (%)	16	17	15
Taking fish oil supplement, at least periodical intake, the previous year (%)	31	29	32
Energy intake 7-d food diary, MJ/day (95% CI)	9.5 (9.0;9.9)	10.7 (9.9;11.4)	8.5 (8.1;9.0)
Energy intake 24-h dietary recall, MJ/day (95% CI)	11.6 (11.0;12.3)	13.1 (11.9;14.2)	10.6 (9.0;11.2)

Note: Values are descriptive summaries of the study population. No formal statistical tests were performed. 7dFD, 7-day food diary; 2×24hDR, two 24-h dietary recalls; CI, confidence interval; BMI, body mass index. Short education: no education after school, vocational training, shorter courses, and short-term higher education.

**Table 5:** Correlations between whole-blood *n*-3 PUFA biomarkers and estimated dietary intake.

Fatty Acid	7dFD All	7dFD Women	7dFD Men	2×24hDR All	2×24hDR Women	2×24hDR Men
N	120	68	52	120	68	52
Sum *n*-3 fatty acids	0.25 **	0.27 *	0.25	0.15	0.03	0.20
Sum *n*-3 fatty acids incl. supplements	0.42 ***	0.42 ***	0.40 **	0.27 **	0.22	0.30 *
Sum *n*-3 fatty acids MSM				0.24 **	0.07	0.19 *
Sum *n*-3 fatty acids MSM incl. supplements				0.43 **	0.39 **	0.38 **
EPA C20:5*n*-3	0.35 **	0.27 *	0.45 ***	0.10	−0.13	0.36 *
EPA C20:5*n*-3 incl. supplements	0.51 ***	0.50 ***	0.56 ***	0.35 ***	0.25 *	0.50 ***
EPA C20:5*n*-3 MSM				0.13	−0.06	0.32 *
EPA C20:5*n*-3 MSM incl. supplements				0.36 **	0.28 *	0.48 **
DHA C22:6*n*-3	0.44 ***	0.47 ***	0.42 **	0.21 *	0.11	0.31 *
DHA C22:6*n*-3 incl. supplements	0.55 ***	0.57 ***	0.53 ***	0.38 ***	0.35 **	0.40 **
DHA C22:6*n*-3 MSM				0.23 **	0.15	0.26 **
DHA C22:6*n*-3 MSM incl. supplements				0.26 *	0.23 *	0.34 *
EPA + DHA	0.46 ***	0.46 ***	0.46 ***	0.17	0.01	0.30 *
EPA + DHA incl. supplements	0.59 ***	0.61 ***	0.58 ***	0.37 ***	0.32 **	0.42 **
EPA + DHA MSM				0.27 **	0.08	0.38 **
EPA + DHA MSM incl. supplements				0.38 ***	0.29 *	0.45 **
Total fish intake	0.35 **	0.39 **	0.32 **	0.27 *	0.08	0.44 **
Total fish intake MSM incl. FPQ				0.28 *	0.09	0.40 **
Lean fish intake	0.12	0.18	0.09	0.16	−0.02	0.34 *
Fatty fish intake	0.43 ***	0.44 ***	0.40 **	0.23 *	0.15	0.27 *

Note: Values are partial Spearman’s rank correlation coefficients. Blood fatty acids were expressed as a weight percentage of total fatty acids. Dietary fatty acid intakes were expressed as percentage of total fat intake. MSM was used to adjust 2×24hDR estimates for intra-individual variation, including age, sex, and age × sex as covariates; * *p* < 0.05, ** *p* < 0.01, *** *p* < 0.001. 7dFD, 7-day food diary; 2×24hDR, two 24-h dietary recalls; MSM, Multiple Source Method; EPA, eicosapentaenoic acid; DHA, docosahexaenoic acid; PUFA, polyunsaturated fatty acids.

**Table 6:** Estimating usual intakes of fish from 7dFD (g/10MJ), 2×24hDR (g/10 MJ), and FPQ. For 2×24hDR using the MSM method without and with using information from a FPQ to estimate usual intake (based on interpreted intake).

Method	Consumers	Mean Fish Intake	1	10	25	50	75	95
7dFD	106 (88%)	29	0	0	7	20	44	84
FPQ using standard portions.	117 (97%)	45	0	16	26	38	54	108
2×24hDR	76 (63%)	32	0	0	0	14	47	134
2×24hDR MSM, default all consumers	120 (100%)	33	11	15	22	29	42	62
2×24hDR MSM, default assumption 50% consumers	60 (50%)	33	0	0	24	34	44	68
2×24hDR MSM + FPQ	117 (97%)	33	0	16	24	32	42	63

Note: Values are descriptive estimates of fish intake expressed as g/10 MJ. MSM including age, sex, and age × sex as covariates was applied only to the 2×24hDR scenarios indicated in the table. The MSM + FPQ model was considered the primary model-based usual-intake scenario; other MSM scenarios were retained as sensitivity analyses. No formal statistical tests were performed for between-method comparisons in this table. 7dFD, 7-day food diary; 2×24hDR, two 24-h dietary recalls; FPQ, Food Propensity Questionnaire; MSM, Multiple Source Method.

**Table 7:** Differences in food intake (g/day) when based on different sampling days and periods by seven-day food diary (Based on registered intake).

Product	Sampling of Days	Proportion Consuming	Population Mean	Consumer Mean	p25	Median	p75
Fish	1 random	61 (51%)	22	43	15	33	60
Ryebread	1 random	79 (66%)	50	76	28	62	97
Coffee	1 random	88 (73%)	321	438	200	354	600
Fish	first	41 (34%)	24	71	35	51	94
Ryebread	first	60 (50%)	56	112	55	88	148
Coffee	first	85 (71%)	353	498	265	400	600
Fish	fourth	44 (37%)	31	83	34	55	128
Ryebread	fourth	58 (48%)	45	94	41	82	123
Coffee	fourth	77 (64%)	324	506	298	400	665
Fish	2 random	73 (61%)	28	46	13	35	67
Ryebread	2 random	74 (62%)	49	80	41	62	97
Coffee	2 random	89 (74%)	326	440	242	375	549
Fish	2 fixed	68 (57%)	24	43	18	36	61
Ryebread	2 fixed	84 (70%)	57	81	41	69	103
Coffee	2 fixed	90 (75%)	351	468	223	400	598
Fish	all 7 days	106 (88%)	26	29	11	23	41
Ryebread	all 7 days	100 (83%)	50	60	22	53	77
Coffee	all 7 days	94 (78%)	323	413	191	357	543

Note: Values are descriptive summaries based on registered intake from the 7dFD. No formal statistical tests were performed. 7dFD, 7-day food diary; p25, 25th percentile; p75, 75th percentile.

**Table 8:** Estimates of different operational definitions of acute intake of fish, ryebread and coffee (g/day) from 7dFD (Based on registered intake).

(a) 7dFD, acute food consumption for consumers, average on a single day, including all days (also zero intake days)									
**Product**	**Consumers**	**Mean**	**p1**	**p5**	**p25**	**median**	**p75**	**p95**	**p99**
Fish	106 (88%)	29	2	3	11	23	41	70	105
Ryebread	100 (83%)	60	4	6	22	53	77	139	200
Coffee	94 (78%)	413	10	50	191	357	543	1044	1202
(b) 7dFD, acute food consumption for consumers on single days including consumption days only									
**Product**	**Consumers**	**Mean**	**p1**	**p5**	**p25**	**median**	**p75**	**p95**	**p99**
Fish	106 (88%)	72	8	14	40	59	89	169	189
Ryebread	100 (83%)	96	9	25	63	84	112	183	362
Coffee	94 (78%)	455	70	120	245	396	573	1047	1202
(c) 7dFD, acute consumption on single meals with an intake									
**Product**	**Consumers**	**Mean**	**p1**	**p5**	**p25**	**median**	**p75**	**p95**	**p99**
Fish	106 (88%)	100	9	15	45	91	138	227	329
Ryebread	100 (83%)	109	12	26	82	112	123	218	280
Coffee	94 (78%)	531	70	150	312	450	600	1067	1400
(d) 7dFD, maximum intake on one consumption day of one product									
**Product**	**Consumers**	**Mean**	**p1**	**p5**	**p25**	**median**	**p75**	**p95**	**p99**
Fish	106 (88%)	108	9	15	46	94	141	258	350
Ryebread	100 (83%)	139	12	26	82	122	165	280	446
Coffee	94 (78%)	659	70	182	400	600	800	1400	1707
(e) 7dFD, meal-weighted daily average intake									
**Product**	**Consumers**	**Mean**	**p1**	**p5**	**p25**	**median**	**p75**	**p95**	**p99**
Fish	106 (88%)	124	10	17	53	108	162	296	402
Ryebread	100 (83%)	151	13	30	94	129	189	287	471
Coffee	94 (78%)	748	80	190	460	664	920	1610	1865

Note: Values are descriptive estimates based on registered 7dFD intake and predefined operational definitions of acute intake. No formal statistical tests were performed. Percentiles describe the observed distribution within the analytical sample and should not be interpreted as population percentiles. 7dFD, 7-day food diary; p1, 1st percentile; p5, 5th percentile; p25, 25th percentile; p75, 75th percentile; p95, 95th percentile; p99, 99th percentile.

**Table 9:** Estimates of different operational definitions of acute intake of fish, ryebread, and coffee (g/day) from 2×24hDR (Based on registered intake).

(a) 2×24hDR, acute food consumption for consumers, average on a single day, including all days (also zero intake days)									
**Product**	**Consumers**	**Mean**	**p1**	**p5**	**p25**	**median**	**p75**	**p95**	**p99**
Fish	72 (60%)	68	8	15	20	41	86	188	398
Ryebread	77 (64%)	70	12	22	35	62	90	158	214
Coffee	93 (78%)	682	75	147	394	670	848	1555	1864
(b) 2×24hDR, acute food consumption for consumers on single days including consumption days only									
**Product**	**Consumers**	**Mean**	**p1**	**p5**	**p25**	**median**	**p75**	**p95**	**p99**
Fish	72 (60%)	106	16	27	40	69	125	313	518
Ryebread	77 (64%)	93	25	43	45	90	120	187	236
Coffee	93 (78%)	722	142	190	454	708	858	1555	1864
(c) 2×24hDR, acute consumption on single meals with an intake									
**Product**	**Consumers**	**Mean**	**p1**	**p5**	**p25**	**median**	**p75**	**p95**	**p99**
Fish	72 (60%)	112	16	30	40	71	130	319	513
Ryebread	77 (64%)	87	25	43	45	90	99	180	202
Coffee	93 (78%)	726	140	202	454	606	848	1660	2471
(d) 2×24hDR, maximum intake on one consumption day of one product									
**Product**	**Consumers**	**Mean**	**p1**	**p5**	**p25**	**median**	**p75**	**p95**	**p99**
Fish	72 (60%)	121	16	30	40	77	160	369	587
Ryebread	77 (64%)	107	25	43	45	90	135	225	347
Coffee	93 (78%)	853	151	217	512	812	1030	1826	2824
(e) 2×24hDR, meal-weighted daily average intake									
**Product**	**Consumers**	**Mean**	**p1**	**p5**	**p25**	**median**	**p75**	**p95**	**p99**
Fish	72 (60%)	138	18	34	46	88	184	425	654
Ryebread	77 (64%)	117	28	43	52	103	142	234	371
Coffee	93 (78%)	978	173	250	589	896	1173	2100	3248

Note: Values are descriptive estimates based on registered 2×24hDR intake and predefined operational definitions of acute intake. No formal statistical tests were performed. Percentiles describe the observed distribution within the analytical sample and should not be interpreted as population percentiles. 2×24hDR, two 24-h dietary recalls; p1, 1st percentile; p5, 5th percentile; p25, 25th percentile; p75, 75th percentile; p95, 95th percentile; p99, 99th percentile.

## Data Availability

In accordance with Danish law and GDPR regulations, the data and analytical scripts used in this study are stored on secure servers at the Technical University of Denmark. Access to these materials requires a Disclosure Declaration and can be granted upon request to applicants who meet the eligibility criteria. Requests for access and Disclosure Declarations may be directed to the Technical University of Denmark via email at: apbj@food.dtu.dk.

## Appendix A

### Formulas

**Acute Food Consumption on Single Days** (Counting All Days, Including Non-Consuming Days):

$$\mathrm{Average}\;\mathrm{intake}\;=\;\Sigma_{i=1}^{n}\;\mathrm{intake}\;\mathrm{on}\;{\mathrm{day}}_{i}/n$$

where:

- *n* = Total number of days observed
- Intake on Day (i) = Amount of food/nutrient consumed on day (i).
- If a food is not registered as consumed on a given day, the intake is assumed to be 0.

**Acute Food Consumption on Single Days of Consumers** (Only Counting Consuming Days):

$$\mathrm{Average}\;\mathrm{intake}\;=\;\Sigma_{i=1}^{c}\;\mathrm{intake}\;\mathrm{on}\;\mathrm{consuming}\;{\mathrm{day}}_{i}/c$$

where:

- c = Number of days with consumption
- Intake on Consuming Day (i) = Amount of food/nutrient consumed on the day with intake.

**Maximum Intake on One Consumption Day** refers to the highest amount of a specific food or nutrient consumed on any single day within the observation period.

Maximum Intake = max (Intake on Day1, Intake on Day2, …, Intake on Day_n_)

where:

- Identify the Intake for Each Day:
  - Record the amount of the specific product consumed on each day of the observation period.
  - If the product was not consumed on a particular day, the intake for that day is 0.
- Determine the Maximum Value:
  - Compare the intake amounts for all the days and identify the maximum value.

**Single Meals Only Counting Meals with an Intake:**

$$\mathrm{Average}\;\mathrm{Intake}\;\mathrm{per}\;\mathrm{Meal}=\Sigma_{i=1}^{m}\;\mathrm{Intake}\;\mathrm{during}\;{\mathrm{Meal}}_{i}/m$$

where:

- m = Number of meals where the food/nutrient was consumed
- Intake during Meal (i) = Amount of food/nutrient consumed during meal (i).

**Weighted Average Intake Across Meals (exploratory operational definition):**

- Calculate the average intake over multiple meals, giving more weight to meals with higher consumption.
- Useful when consumption is irregular, but the aim is to account for high-intake meals more heavily. (e.g., main meals vs. snack, more weight on main meals and more weight on dinner)

$$\mathrm{Weighted}\;\mathrm{average}\;\mathrm{meal}\;\mathrm{intake}=\Sigma_{i=1}^{m}\;w_{i}\;\mathrm{Intake}\;\mathrm{during}\mathrm{meal}\;i$$

where:

- (wᵢ) = Weight assigned to meal i based on expected contribution to intake or another predefined criterion.
- m = Total number of meals observed.
